# Modeling Working Memory in Neurodegeneration: A Focus on EEG Methods

**DOI:** 10.3390/diagnostics15232992

**Published:** 2025-11-25

**Authors:** Yuliya Komarova, Alexander Zakharov, Mariya Sergeeva, Natalia Romanchuk, Tatyana Vladimirova, Igor Shirolapov

**Affiliations:** 1 Neurosciences Research Institute, Federal State Budgetary Educational Institution of Higher Education, Samara State Medical University, Ministry of Healthcare of the Russian Federation, 443099 Samara, Russia; 2 Department of Otorhinolaryngology n.a. Academician I.B. Soldatov, Federal State Budgetary Educational Institution of Higher Education, Samara State Medical University, Ministry of Healthcare of the Russian Federation, 443099 Samara, Russia

**Keywords:** working memory, electroencephalography, mild cognitive impairment, neurodegeneration, machine learning

## Abstract

Working memory is one of the most vulnerable cognitive domains in neurodegenerative diseases. According to the World Health Organization, around 55 million people worldwide were living with dementia in 2021, a number projected to exceed 150 million by 2050. Impairments in working memory occur in 80–90% of patients with Alzheimer’s disease, 40–60% with Parkinson’s disease, and about 50% with frontotemporal dementia. These deficits include reduced information capacity, slower response times, increased errors in manipulation, and difficulties in maintaining information, making them sensitive indicators of progressive decline. This review aims to systematize current approaches to modeling working memory phenotypes using electroencephalography (EEG). It highlights experimental paradigms applied to probe working memory, methods of EEG signal processing and analysis, and the integration of machine learning and neural network models. Particular emphasis is placed on studies achieving high diagnostic accuracy, with classification rates of 85–90% when distinguishing patients with neurodegeneration from healthy participants. Limitations of existing methods, especially EEG variability, are considered. The review concludes by outlining future directions: integration of multimodal EEG data, application of artificial intelligence, and development of digital cognitive biomarkers for hybrid models capable of predicting cognitive decline and advancing clinical translation.

## 1. Introduction

Working memory is a fundamental cognitive process responsible for the temporary storage and manipulation of information necessary for goal-directed behavior, decision-making, and learning. Impairments in working memory are recognized as one of the earliest and most prominent symptoms of neurodegenerative diseases, including Alzheimer’s disease, mild cognitive impairment, and frontotemporal dementia. Elucidating working memory phenotypes and the mechanisms underlying their alteration in pathological aging represents a critical challenge for both fundamental neuroscience and the development of clinically relevant biomarkers [1,2].

The emergence of advanced analytical approaches underscores the necessity not only to record but also to formalize neural activity using computational models. The history of computational models describing the electrical activity of neural tissue spans a century. One of the earliest formalizations was the “integrate-and-fire” model, proposed by L. Lapicque in 1907. This model conceptualized the neuron as an equivalent electrical circuit composed of a capacitor and a leak resistor, thereby describing the summation of postsynaptic potentials and the generation of action potentials [3]. A significant qualitative advancement was achieved by A. Hodgkin and A. Huxley (1952), who introduced a formalized model of the squid giant axon membrane, which became a cornerstone of modern neurophysiology [4]. In the 1960s, W. Rall expanded upon these concepts by proposing the cable theory for dendritic trees, laying the foundation for contemporary multi-compartmental modeling [5,6].

In parallel with biophysical models, systemic methods for brain research have also advanced significantly. Non-invasive techniques for recording bioelectrical activity have gained particular importance, among which electroencephalography (EEG) plays a leading role. Electroencephalography holds a prominent position among cognitive neuroimaging techniques due to its high temporal resolution, relative accessibility, and direct measurement of neuronal activity [7]. Unlike structural or metabolic methods, EEG enables the tracking of oscillatory dynamics, which reflect real-time interactions within neural networks. These properties render EEG one of the most promising tools for investigating cognitive impairments in neurodegenerative disorders.

Over the past two decades, EEG analysis approaches have substantially evolved, progressing from traditional spectral power and coherence analysis to sophisticated multivariate models incorporating entropy measures, functional connectivity metrics, and machine learning methods [8,9]. Particular emphasis is placed on the identification of working memory phenotypes—stable neurodynamic patterns that reflect both compensatory reorganization and pathological changes within cognitive networks. The characterization of these phenotypes enables early diagnosis, predicts cognitive decline, and differentiates subtypes of neurodegenerative diseases.

Recent studies demonstrate that integrating EEG metrics with classification and modeling algorithms yields high diagnostic accuracy, with approaches such as event-related potential (ERP) analysis, event-related oscillation (ERO) analysis, or deep neural network architectures exceeding 90% accuracy in binary and multiclass classification tasks [10]. Importantly, these methods also facilitate the interpretation of EEG phenotypes in terms of underlying neurobiological mechanisms, ranging from synaptic plasticity deficits and excitatory-inhibitory imbalance to the disintegration of prefrontal–hippocampal and parietal networks [11,12]. Thus, the high temporal resolution of EEG complements classical computational models of neural activity, creating a unified continuum—from membrane equations to network dynamics and cognitive phenotypes.

## 2. Materials and Methods

**Figure 1:**
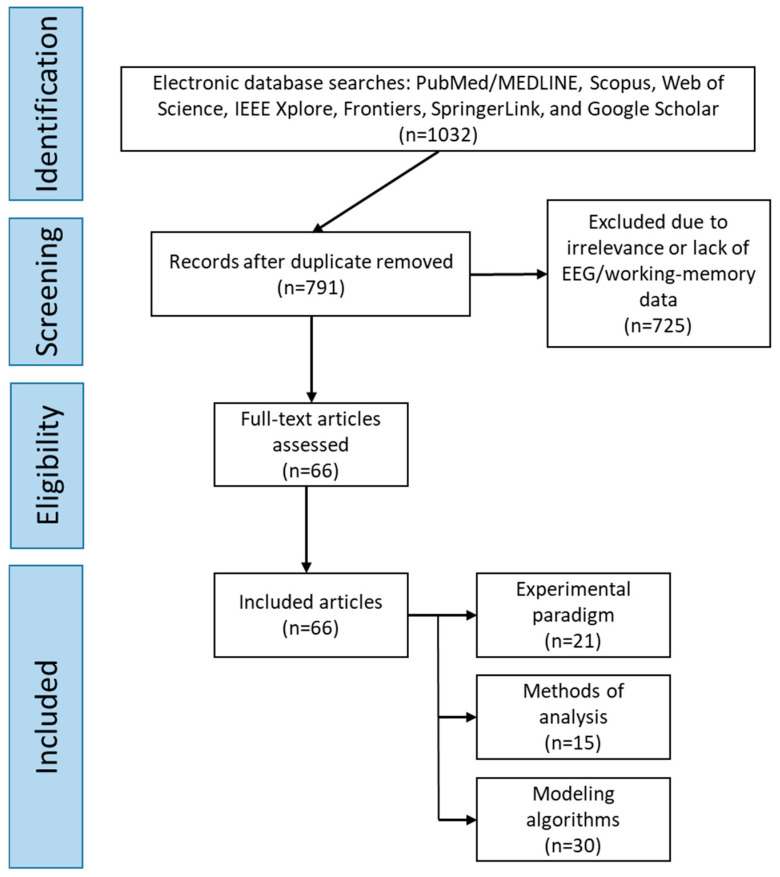
Flow Diagram of Literature Search and Selection Criteria.

This scoping review was conducted in accordance with the PRISMA-ScR (Preferred Reporting Items for Systematic Reviews and Meta-Analyses extension for Scoping Reviews) guidelines. A review protocol was developed a priori based on PRISMA-P principles and included predefined inclusion and exclusion criteria, as well as detailed procedures for study selection, data extraction, and data synthesis (Figure 1).

The research question was formulated using the PCC (Population, Concept, Context) framework:

- Population: Adult participants (≥18 years), including healthy controls (HC), individuals with mild cognitive impairment (MCI), Alzheimer’s disease (AD), frontotemporal dementia (FTD), and other forms of pathological aging.
- Concept: Analysis of EEG-based working memory phenotypes employing machine learning and deep learning methods. Eligible studies reported spectral power measures, coherence, entropy, event-related potentials (ERP), event-related oscillations (ERO), and metrics of functional connectivity.
- Context: Laboratory cognitive paradigms, clinical investigations of patients with neurodegenerative disorders, and experimental studies involving working memory tasks.

Inclusion criteria:

- Original empirical studies (randomized controlled trials, cross-sectional, cohort, and longitudinal designs);
- Publications in peer-reviewed journals;
- Availability of EEG features related to working memory;
- Application of classification or modeling approaches.

Exclusion criteria:

- Studies lacking EEG data or not involving a working-memory cognitive paradigm;
- Studies involving participants under 18 years of age;
- Publications without accessible full texts.

The literature search was conducted in the following databases: PubMed/MEDLINE, Scopus, Web of Science, IEEE Xplore, Frontiers, SpringerLink, and Google Scholar.

Search queries included combinations of the following keywords:

- “EEG” AND “working memory” AND “neurodegeneration” AND “Alzheimer’s disease”;
- “EEG” AND “working memory” AND “mild cognitive impairment”;
- “event-related oscillations” OR “event-related potentials” AND “cognitive impairment”;
- “machine learning” OR “deep learning” AND “EEG” AND “working memory”;
- “neurodegeneration” AND “EEG phenotypes” AND “connectivity.”

Search Results:

- Total records identified: 1032;
- Duplicates removed: 241;
- Excluded due to irrelevance or lack of EEG/working-memory data: 725;
- Included in the final analysis: 66 studies.

## 3. Cognitive Impairments and the Role of Working Memory in the Diagnosis of Neurodegenerative Processes

Cognitive impairments represent one of the most significant medical and social challenges in contemporary society. Their prevalence and progressive nature not only reduce patients’ quality of life but also create a substantial socio-economic burden, associated with long-term care needs, reduced workforce participation, and increased healthcare costs [13]. Among the various cognitive domains, working memory—a system responsible for the short-term retention and active manipulation of information—has received particular attention from researchers [14,15]. Dysfunctions in this component are considered one of the earliest and most sensitive markers of neurodegenerative processes, as working memory integrates attention, executive functions, and long-term memory [16].

According to the World Health Organization, approximately 55 million people worldwide were living with dementia in 2021, and this number is projected to exceed 150 million by 2050. The highest prevalence of working memory impairments is observed in Alzheimer’s disease, affecting 80–90% of patients and manifesting as both reduced retention capacity and errors in updating and processing information [17]. In Parkinson’s disease, working memory deficits occur in 40–60% of cases, often accompanied by executive dysfunctions resulting from disrupted fronto-striatal connectivity [18]. In frontotemporal dementia, roughly 50% of patients exhibit working memory impairments, primarily involving deficits in retention and updating, while short-term sensory memory remains relatively preserved [14].

Clinically, these impairments manifest as reduced working memory capacity, prolonged reaction times, increased errors in information manipulation, and difficulties in maintaining information over time. Such characteristics confer a key diagnostic and prognostic role to working memory, as its dysfunction may indicate not only the presence of cognitive deficits but also their progression. Consequently, studying working memory in neurodegenerative diseases is of both scientific and clinical significance, offering opportunities for early diagnosis, disease monitoring, and the development of cognitively targeted therapeutic interventions [19,20].

Because behavioral assessments alone do not fully capture the neurobiological mechanisms underlying cognitive impairments, methods that directly measure brain activity are of particular importance [21]. In this context, working memory serves as a convenient model system: its relatively simple structure and well-established cognitive paradigms allow the identification of specific neurophysiological markers. Among available research methods, electroencephalography (EEG) holds particular promise, providing high temporal resolution and enabling the tracking of neuronal dynamics underlying information retention and updating [22].

## 4. Experimental Paradigms of Working Memory

Implementing this approach requires experimental tasks that allow manipulation of cognitive load and elicit reproducible EEG changes. Among these, n-back working memory paradigms are the most widely used, enabling systematic investigation of information retention and updating under varying levels of difficulty. Contemporary studies employ 1-, 2-, and 3-back tasks to stratify participants and identify sensitive EEG markers [23,24]. Transitioning from 1-back to 2-back in patients with mild cognitive impairment (MCI) and Alzheimer’s disease results in pronounced reductions in theta-gamma coupling, accompanied by decreased desynchronization and lower amplitudes of event-related potentials [25]. These changes are also reflected in attenuated ERD/ERS responses (event-related (de)synchronization) and reduced ERP amplitudes (event-related potentials), highlighting their potential as early diagnostic biomarkers [26].

Another classical paradigm is the Sternberg task, in which participants memorize a set of stimuli and subsequently identify whether a presented test stimulus belongs to the original set. Manipulating the set size allows precise control over working memory load and facilitates tracking the dependence of neurophysiological responses on memory demands. Studies indicate that reduced high-frequency oscillatory activity particularly in the beta band—which significantly differs between Alzheimer’s patients and age-matched healthy controls—may reflect the neuronal activity underlying early-stage visual working memory deficits in Alzheimer’s disease [27].

Delayed match-to-sample paradigms represent another important approach, differentiating processes of encoding, retention, and comparison. Participants are shown a target stimulus, followed by comparison stimuli after a variable delay, requiring a match/non-match judgment. This design separates the maintenance phase from active comparison, which is particularly valuable for investigating working memory dysfunctions. Recent findings demonstrate that frontal ERP components elicited in these tasks can predict MCI development prior to clinical symptom onset, highlighting their prognostic value [28,29].

Increasing attention is being given to multimodal working memory paradigms, which combine stimuli across sensory modalities, such as auditory and visual, to probe integrative mechanisms of cognitive load. Working memory has been conceptualized in terms of the transition from resting state to task execution, reflected in the Working Memory Task-Induced EEG Response (WM-TIER) index [10]. Functional connectivity measures recorded during multimodal tasks, particularly phase synchronization indices, provide higher classification accuracy for MCI and Alzheimer’s patients compared to resting-state EEG, emphasizing the critical role of cognitive load in revealing pathological patterns. In this context, multimodal machine learning frameworks integrating EEG with other modalities and synchronous behavioral metrics demonstrate high efficiency and offer new avenues for improved diagnosis and patient stratification [30,31].

## 5. EEG Analysis Methods

The neuronal cell membrane functions as an electrical dielectric, making it impossible to record intracellular currents using scalp electrodes. Instead, EEG captures currents flowing through the extracellular space, which propagate through brain tissue to electrodes on the scalp, a phenomenon known as “volume conduction” [32]. To obtain meaningful EEG signals, the activity of a large number of neurons must be synchronized, generating electrical dipoles. These dipoles have a specific orientation: the negative pole is located closer to the cortical surface layers, while the positive pole lies deeper.

The summation of small dipoles creates currents of sufficient amplitude to be detected at the scalp, allowing EEG to visualize brain electrical activity and analyze patterns such as the alpha rhythm or epileptiform discharges [33]. However, accurate identification and analysis of EEG signals require a deep understanding of their complex theoretical properties and extraction of relevant features for specific tasks. EEG signals present significant challenges due to susceptibility to noise, resulting in a low signal-to-noise ratio, as well as their nonlinearity and deviation from normal distributions, distinguishing them from conventional signals [34]. Individual factors such as age, psychological state, and testing conditions can also introduce substantial variability [35]. These unique characteristics necessitate diverse analytical methodologies to extract task-relevant information effectively [36].

Particularly promising are combined approaches that not only record but also modulate brain activity. One such approach is the combination of transcranial magnetic stimulation (TMS) with EEG, enabling non-invasive, direct investigation of neuronal excitability and functional connectivity of targeted cortical regions, making it valuable for both physiological studies and the diagnosis of pathological states [37].

Unlike methods measuring indirect proxies of brain activity, EEG records summed postsynaptic potentials in real time, providing millisecond-level temporal precision [38]. This allows not only analysis of local neurodynamic features but also modeling of interactions across brain regions. Notably, EEG signals can be decomposed into neuronal oscillations or rhythms, which constitute a fundamental mechanism for information transfer and interregional coordination of cognitive processes [39,40,41].

In the context of working memory and cognitive aging, analysis of EEG rhythmic activity and network characteristics has become central. Early systematic studies by Jiang et al. [42,43] demonstrated increased spectral power and coherence in θ, α, and β bands in patients with mild cognitive impairment during both resting state and working memory tasks. These findings were interpreted as reflecting compensatory network reorganization and reduced efficiency of functional connectivity, establishing a methodological basis for further studies.

Subsequent research has shifted towards more sophisticated models, such as the WM-TIER approach, which normalizes task-related EEG metrics against the resting-state baseline. This framework incorporates three classes of features: relative spectral power, spectral coherence, and the Filter Bank Phase Lag Index to construct a functional network representation of neurodynamics. This methodology enhances the sensitivity and specificity of the technique, as evidenced by its superior accuracy in classifying Alzheimer’s disease and MCI compared to the use of either resting-state or task-elicited EEG data alone [10].

Entropy-based methods and hybrid preprocessing approaches have also emerged, combining ICA and wavelet denoising with subsequent computation of spectral, coherence, and entropy measures (spectral, approximate, permutation entropy) [44,45], enabling differentiation between vascular dementia, post-stroke MCI, and healthy participants. Later refinements integrated feature selection, channel optimization, and dimensionality reduction, improving classification accuracy [46].

Research has also focused on EEG dynamics and variability. Trinh et al. [47] introduced an intra-subject variability metric, comparing spectral power before and after a cognitively demanding delayed match-to-sample task, which serves as an indicator of cognitive network stability and enhances MCI classification with machine learning algorithms. Other studies examined auditory ERP components, extracting over 590 features—including temporal, time-frequency, and morphological ERP characteristics—from a single Fpz channel. Machine learning methods (SVM, logistic regression) achieved high accuracy in classifying patients with mild cognitive impairment, demonstrating the diagnostic potential of minimally invasive recording protocols [48].

Liao et al. [9] highlighted the diagnostic value of event-related oscillations (EROs) in θ, α, and γ bands. Machine learning analyses showed that ERO metrics are highly informative for differentiating preclinical Alzheimer’s disease from healthy aging, confirming their promise as biomarkers of cognitive decline.

## 6. Classification and Modeling Algorithms

Although the concept of “networks” has been discussed in neuroscience for many years, recent advances in applied mathematics have opened new avenues for quantitative analysis of these network structures. Research has demonstrated that normal brain function depends on the interaction of distinct regions, which form a complex large-scale network with interconnected, densely linked hubs that serve as a critical foundation for effective communication between neurons [49]. Notably, the human cerebral cortex is characterized by a small-world organization [50,51]. This architectural topology provides an optimal balance between local, specialized information processing—reflected in a high clustering coefficient—and global integration, facilitated by short path lengths connecting distant network nodes, which promotes efficient information propagation. From a computational standpoint, this organization is considered highly efficient, as it supports both the modular specialization of functional networks and their coordinated interaction. In neurodegenerative pathologies, particularly Alzheimer’s disease, a disruption of this optimal structure is observed [52]. The network shifts towards a pathological state characterized by either excessive localization—fragmenting into isolated clusters—or excessive randomization of connections. In both scenarios, this leads to a significant decline in the efficiency of cognitive processing [53]. Synaptic connectivity in the cortex influences computational network models, and pathology in Parkinson’s disease affects these synaptic mechanisms [54]. Quantitative assessment of network physiology in the context of Parkinson’s pathology and cognitive decline offers a promising approach for understanding abnormal interactions among multiple cortical regions that cannot be explained by traditional analyses.

Algorithms applied to EEG data have undergone significant evolution in recent years, transitioning from traditional machine learning methods to hybrid and deep architectures that provide both high accuracy and deeper insight into neurodynamics during cognitive aging. While early studies primarily used SVM, k-NN, or logistic regression with limited feature sets, contemporary approaches integrate advanced feature extraction with deep learning models, enabling multiclass classification and cognitive load prediction [55,56].

For example, Sridhar et al. employed a deep neural network (DNN) based on Ensemble Empirical Mode Decomposition (EEMD) to extract informative intrinsic mode functions (IMFs) and construct a model for predicting cognitive load, achieving an average accuracy of 97.62% in distinguishing healthy controls from patients with mild cognitive impairment [57]. Similarly, the CNN-LSTM hybrid architecture DeepADNet combines convolutional layers for spatial patterns and recurrent layers for temporal dynamics, enabling high accuracy in multiclass classification of neurodegeneration stages [58].

Another line of research involves transforming spectral and time–frequency EEG signals into image representations. One approach segments one-dimensional EEG signals into augmented temporal windows for analysis with a modified DPCNN, achieving ~97.1% accuracy and an F1 score of 97.11% in three-class classification (HC, MCI, AD) [59]. Continuous wavelet transforms combined with AlexNet architectures have further increased accuracy to 98.9% [60].

Deep convolutional architectures such as ResNet, DeepConvNet, and EEGNet have also been applied to model nonlinear dependencies. ResNet-18 applied to coherence matrices reached 93.4% accuracy in MCI classification and 98.5% in differentiating AD from healthy participants, highlighting the informativeness of EEG network metrics [61]. Lighter architectures targeting functional connectivity, such as shallow neural networks analyzing amplitude envelope correlation (AEC), achieved 94.5% accuracy in distinguishing Alzheimer’s disease from frontotemporal dementia, outperforming both classical algorithms and CNNs [62].

These advancements reflect a trend toward balancing model complexity and EEG feature informativeness. Table 1 summarizes key recent studies, including experimental paradigms, preprocessing and EEG analysis methods, and machine learning algorithms, illustrating the evolution from early descriptive studies to modern hybrid and deep models achieving 90–98% classification accuracy in both binary and multiclass tasks.

In another study, EEG data and source localization methods were used to analyze brain activity. In one approach, the cloud platform iSyncBrain [63] was employed for signal preprocessing, spectral analysis, and source localization using sLORETA on the Colin 27 model with parcellation according to the Desikan–Killiany atlas (68 ROIs). Power spectral density was computed using the fast Fourier transform with a 0.25 Hz step across delta, theta, alpha 1–2, beta 1–3, and gamma bands, with particular emphasis on alpha and beta rhythms. Both absolute and relative power were evaluated, and electrodes were grouped into five anatomical regions. Network analysis was performed using graph theory, including global metrics (global efficiency, path length, clustering coefficient, small-worldness) and local metrics (local efficiency, node degree, centrality), allowing characterization of the integrative and segregative properties of the networks.

In parallel, in the study [64], source localization was conducted using the boundary element method based on three-dimensional surfaces of the Montreal Neurological Institute brain template. Frequency analysis was performed at the sensor level for delta (0.5–4 Hz), theta (4–8 Hz), alpha (8–13 Hz), and beta (13–30 Hz) bands using Fourier transformation. Current source density was estimated with eLORETA in the 200–700 ms interval, reflecting late responses in Parkinson’s disease. Sources were mapped to the Brodmann atlas (42 ROIs), with eight regions selected for functional connectivity and spectral power analysis. Functional connectivity was calculated using the imaginary part of coherence (FieldTrip) to minimize artifacts, and network topology was evaluated using degree, clustering coefficient, and global efficiency metrics for significant connections among 16 ROIs with a threshold of 0.07 and correction for false discovery rate. The neurophysiological basis of memory involves activity-dependent changes in synaptic efficacy, such as long-term potentiation (LTP) and long-term depression (LTD) [65].

However, despite the reported high diagnostic accuracy (85–98%), the approaches summarized in Table 1 require critical appraisal concerning their methodological rigor and clinical applicability. A key challenge is the persistent risk of overfitting in machine learning models, particularly when working with small and imbalanced datasets [66]. For instance, the study by Sridhar et al. [57], which reported an accuracy of 97.62%, was conducted on a cohort of only 18 healthy subjects, raising concerns about the reproducibility of the results in independent cohorts [67]. Mitigating this risk necessitates robust validation strategies, including nested cross-validation and, most critically, testing on completely independent datasets acquired from different clinical centers [68].

A further fundamental limitation is the high inter-individual variability of EEG signals, attributable to anatomical differences (e.g., skull thickness, gyrification), pharmacological effects, comorbid conditions, and fluctuations in functional state [69]. To account for this variability, normalization techniques (e.g., the use of relative spectral power) and data aggregation from large-scale, multi-center studies are employed, enabling models to discern generalizable patterns rather than adapting to noise.

A significant barrier to clinical implementation is the limited interpretability of complex deep learning models, such as hybrid CNN-LSTM architectures [58]. Despite their high accuracy metrics, their decision-making processes often remain a “black box” to clinicians, creating a trust deficit and hindering integration into diagnostic workflows. A promising avenue to address this issue is the development of Explainable Artificial Intelligence (XAI) methods, particularly the use of saliency maps, which help identify the specific EEG features that contributed most significantly to the classification outcome [70].

## 7. Conclusions

The systematic analysis of contemporary methods for modeling working memory phenotypes using EEG in neurodegenerative diseases allows several key conclusions to be drawn and highlights promising directions for future research. Accumulated evidence convincingly indicates that working memory impairments—one of the earliest and most sensitive markers of cognitive decline in conditions such as Alzheimer’s disease, Parkinson’s disease, and frontotemporal dementia—have clear neurophysiological correlates detectable via EEG.

From a neurobiological perspective, these EEG phenotypes reflect profound disruptions at both the cellular and network levels. Reduced amplitude of late event-related potential components and attenuated induced theta and gamma activity in frontoparietal networks appear to result directly from synaptic dysfunction and loss of synaptic plasticity caused by pathological accumulation of tau protein and beta-amyloid. Attenuated event-related desynchronization (ERD) in the alpha and beta bands may indicate deficits in top-down control and impaired inhibition mediated by GABAergic interneurons. Altered functional connectivity patterns, particularly reductions in small-world network efficiency, reflect progressive disintegration of key hubs such as the prefrontal cortex and hippocampus, disrupting the synchronization necessary for successful encoding, maintenance, and manipulation of information in working memory.

The methodological toolkit for studying these processes has expanded considerably, ranging from classical paradigms (n-back, Sternberg) to complex multimodal tasks that reveal deficits under cognitive load. Modern analytical approaches—including functional connectivity analysis, time–frequency decomposition, and entropy measures—allow quantitative assessment of these disruptions. A major breakthrough has been the integration of machine learning algorithms and deep neural networks (e.g., CNN, LSTM, and hybrid architectures), achieving exceptionally high accuracy (up to 85–98%) in differential diagnosis and patient stratification, effectively transforming raw EEG data into powerful digital biomarkers.

Despite significant progress substantial limitations remain, primarily due to high interindividual variability in EEG signals caused by anatomical differences, aging, and distinct patterns of neurodegeneration. Overcoming these challenges requires further standardization of recording and preprocessing protocols.

Future directions include the integration of multimodal data (e.g., TMS-EEG) to relate surface electrical activity to structural and metabolic changes, providing a more complete pathophysiological explanation of observed phenomena. The development of hybrid artificial intelligence models combining EEG analysis with clinical and genetic data may enable personalized prediction of individual trajectories of cognitive decline at preclinical stages. In this way, the combination of advanced EEG technologies and computational approaches brings us closer to early preclinical diagnosis and the creation of objective tools for monitoring therapeutic interventions, a central goal of modern neurology and gerontology.

## Figures and Tables

**Table 1:** Key EEG-based Classification Studies of Cognitive Impairment Using Machine Learning and Deep Learning Approaches.

Authors	Preprocessing Method	Feature Characteristics	Architecture/Algorithm	Sample Size	Classification Task	Accuracy
Sridhar et al. [57]	EEMD (Ensemble Empirical Mode Decomposition)	Intramodal Functions (IMFs)	DNN	18 HC	HC vs. MCI	97.62%
Thi Kieu Khanh Ho [58]	ERSP extraction (event-related spectral perturbation)	Time–frequency patterns (delta–theta–alpha ranges)	CNN + LSTM (hybrid architecture)	Oddball: 63 (23 HC, 17 aAD, 23 pAD); N-back: 36 (13 HC, 11 aAD, 12 pAD)	Neurodegeneration stage classification	Oddball: 71.95% ± 0.019 (raw), 75.95% ± 0.017 (oversampled); N-back: 69.40% ± 0.003 (raw), 73.70% ± 0.010 (oversampled)
Wei Xia [59]	Overlapping sliding windows to augment the one-dimensional EEG	Temporal signal segments; sample augmentation	Deep Pyramid CNN (DPCNN)	100 (49 AD, 37 MCI, 14 HC)	HC, MCI, AD	97.10%; F1: 97.11%
Cameron J Huggins [60]	Artifact cleaning; CWT (Morse mother wavelet)	Time–frequency maps (0–600), topographic images according to 10–20 system; final dataset: 16,197 images	AlexNet	141 (52 AD, 37 MCI, 52 HA)	AD vs. MCI vs. HA (healthy ageing)	98.9%
Feng Duan [61]	Frequency-domain analysis (θ, low α, high α); functional connectivity	Global metrics (network resilience), connectivity metrics, node versatility; LOFC bands	ResNet-18	Datasets: MCI and mild AD (exact n not specified)	HC vs. MCI; HC vs. mild AD	MCI: 93.42% (avg.), up to 98.33% (best); mild AD: 98.54% (avg.), up to 100% (best)
Zaineb Ajra [62]	Signal cleaning; extraction of spectral–temporal features and functional connectivity (multiple thresholds)	Functional connectivity	Shallow NN	88 participants (36 AD, 23 FTD, 29 HC)	AD vs. FTD vs. HC	94.54%

## Data Availability

No new data were created or analyzed in this study. Data sharing is not applicable to this article.
